# Bee Venom in Wound Healing

**DOI:** 10.3390/molecules26010148

**Published:** 2020-12-31

**Authors:** Anna Kurek-Górecka, Katarzyna Komosinska-Vassev, Anna Rzepecka-Stojko, Paweł Olczyk

**Affiliations:** 1 Department of Community Pharmacy, Faculty of Pharmaceutical Sciences in Sosnowiec, Medical University of Silesia in Katowice, Kasztanowa 3, 41-200 Sosnowiec, Poland; polczyk@sum.edu.pl; 2 Department of Clinical Chemistry and Laboratory Diagnostics, Faculty of Pharmaceutical Sciences in Sosnowiec, Medical University of Silesia in Katowice, Jedności 8, 41-200 Sosnowiec, Poland; kvassev@sum.edu.pl; 3 Department of Drug Technology, Faculty of Pharmaceutical Sciences in Sosnowiec, Medical University of Silesia in Katowice, Jedności 8, 41-200 Sosnowiec, Poland; annastojko@sum.edu.pl

**Keywords:** bee venom, wound healing, biological properties, molecular mechanism

## Abstract

Bee venom (BV), also known as api-toxin, is widely used in the treatment of different inflammatory diseases such as rheumatoid arthritis or multiple sclerosis. It is also known that BV can improve the wound healing process. BV plays a crucial role in the modulation of the different phases of wound repair. It possesses anti-inflammatory, antioxidant, antifungal, antiviral, antimicrobial and analgesic properties, all of which have a positive impact on the wound healing process. The mentioned process consists of four phases, i.e., hemostasis, inflammation, proliferation and remodeling. The impaired wound healing process constitutes a significant problem especially in diabetic patients, due to hypoxia state. It had been found that BV accelerated the wound healing in diabetic patients as well as in laboratory animals by impairing the caspase-3, caspase-8 and caspase-9 activity. Moreover, the activity of BV in wound healing is associated with regulating the expression of transforming growth factor (TGF-β1), vascular endothelial growth factor and increased collagen type I. BV stimulates the proliferation and migration of human epidermal keratinocytes and fibroblasts. In combination with polyvinyl alcohol and chitosan, BV significantly accelerates the wound healing process, increasing the hydroxyproline and glutathione and lowering the IL-6 level in wound tissues. The effect of BV on the wounds has been proved by numerous studies, which revealed that BV in the wound healing process brings about a curative effect and could be applied as a new potential treatment for wound repair. However, therapy with bee venom may induce allergic reactions, so it is necessary to assess the existence of the patient’s hypersensitivity to apitoxin before treatment.

## 1. Introduction

Wound healing is a complex process consisting of four overlapping phases: hemostasis and the formation of a clot, inflammation, proliferation, which involves the biosynthesis of the extracellular matrix (ECM), epithelialization and angiogenesis and the last stage—tissue remodeling [1,2,3]. The first stage starts after an injury appears. The narrowing of the vessels is caused by serotonin, thromboxane A2 and adrenaline. The platelets undergo adhesion, aggregation and activation. After 24 h from the injury, a temporary matrix in wound is created. The aggregated platelets release from alfa-granules as growth factors, namely platelet derived growth factor (PDGF), transforming growth factor α (TGF-α), transforming growth factor β (TGF-β), fibroblast growth factor (bFGF), insulin-like growth factor 1 (IGF-1), vascular endothelial growth factor (VEGF) [1,4,5]. PDGF and TGF-β recruit monocytes and neutrophils and initiate an inflammatory response and start the second stage of the healing process—the inflammatory phase. TGF-α, bFGF and VEGF activate endothelial cells to angiogenesis [1,4].

The inflammatory phase begins after 24 h from injury and lasts for up to 48 h. This stage is characterized by vessels widening and increasing their permeability. The process is supported by histamine, kinins, prostaglandins, leukotrienes, nitrogen oxide, hydrolases and reactive oxygen species (ROS). Neutrophils and macrophages clean the wound bed and release cytokines and growth factors, which are responsible for initiating the healing process. Neutrophiles releasing IL-1 and TNF-α quicken the inflammatory process. After two or three days, neutrophils undergo apoptosis and are replaced by monocytes. Due to the influence of TGF-β and the products of the degradation of fibrin and fibronectin, monocytes are transformed into macrophages. Macrophages take part in phagocytosis, clean wounds from bacteria and dead tissue and release proinflammatory cytokines such as IL-1, IL-6, Il-8, TNF-α and growth factors like PDGF, TGF-α, TGF-β, IGF-1,FGF [6,7,8]. Growth factors regulate the inflammatory process. Additionally, cytokines and growth factors activate fibroblasts and epithelial cells to the third stage of healing, i.e., proliferation. The stage of proliferation is connected with the reconstruction of damaged tissue by the proliferation of fibroblasts, epithelial cells and keratinocytes. Fibroblasts excrete: IGF-1, bFGF, TGF-β, PDGF, EGF. Epithelial cells produce: VEGF, bFGF, PDGF. Keratinocytes excrete TGF-α, TGF-β, KDAF (keratinocyte-derived autocrine growth factors). The secreted mediators take part in the biosynthesis of the extracellular matrix, epithelialization and angiogenesis. About four days after injury, the granulation tissue consists of collagen (type I and type III), elastin, proteoglycans, glycosaminoglycans and noncollagenous proteins. Initially, the matrix contains a great amount of hyaluronic acid and fibronectin, however, eventually hyaluronic acid is replaced by collagen. The matrix of granulation tissue is enriched in heparan-sulfate proteoglycans and chondroitin–dermatan proteoglycans [2,9].

The granulation tissue replaces the dermis and transforms into a scar during the next phase—remodeling. Epithelialization is based on the renewal of the epithelium after damage. EGF, KGF and TGF-α influence the migration and proliferation of epithelial cells. The huge role in the epithelialization process is also played by the matrix metalloproteinases such as MMP-2 (gelatinase-A) and MMP-9 (gelatinase-B) and MMP-I (interstitial collagenase), which are responsible for the degradation of collagen type IV, VII. Endothelial progenitor cells derived from hematopoietic stem cell linage take part in neovascularization. Growth factors regulate the inflammatory process, play a chemotactic role for neutrophils, monocytes, macrophages, fibroblasts, keratinocytes and initiate angiogenesis and the formation of an extracellular matrix [10]. Angiogenesis is an important phase of the healing process as it creates new blood vessels. Angiogenesis stimulates the tissue repair process. It is stimulated by: bFGF, TGF-β, TNF-α and VEGF [2].

The proliferation phase is connected with the activity of fibroblasts and the formation of new blood vessels and extracellular matrix. After the proliferation phase, the process of remodeling begins. In the last-fourth phase, the extracellular matrix is rebuilt and the production of collagen type I increases. Myofibroblasts which occur in the wound close the wound. The scar is the end result of fibroblast apoptosis and extracellular matrix degradation [11].

Disorders in the functioning of stem cells and in the synthesis and degradation of growth factors may lead to an impaired wound healing process as well as to hypertrophic scar creation.

Bee venom is a promising component of wound dressing due to its anti-inflammatory effect and enhanced wound repairing capacity. It was found to significantly accelerate the healing of diabetic wounds [12,13]. The slowed wound repair process is connected with hypoxia [12,14]. Insufficient recruitment of macrophages and neutrophiles into the wound environment delay the start of the inflammatory phase and in this way impair the wound healing process. On the other hand, hypoxia amplify the early inflammatory response through the prolonged release of inflammatory cytokines. Moreover, hyperglycemia increases oxidative stress and causes many dysfunctions such as defective T cell immunity, defects in phagocytosis and dysfunctions of fibroblasts. The accumulation of reactive oxygen species (ROS) increases the cellular damage and impairs neovascularization. In addition, faster apoptosis, reduced angiogenesis, and impaired lymphangiogenesis influence the impaired healing process [12,15].

The impaired diabetic wound healing is characterized by a lower collagen production, downregulated expression of TGF-β, VEGF, as well as the upregulated expression of ATF-3 (activating transcription factor 3), iNOS, and an increased activity of caspase-3, caspase-8 and caspase-9. Interestingly, during hypoxia, the mobilization of endothelial progenitor cells (EPCs) is activated by VEGF secreted by macrophages, fibroblasts and epithelial cells, while in diabetic wounds there is a VEGF deficiency. Thus, impaired endothelial progenitor cells (EPCs) mobilization is responsible for endothelial progenitor cell deficiency [11].

## 2. Chemical Composition of Bee Venom

Bee venom is a liquid mixture which contains 88% of water and only 0.1 g of dry weight of a complex mixture of peptides, enzymes and nonpeptide components in one drop [16]. The composition of dry and fresh bee venom differs in contents of volatile components but the biological properties are similar. The composition of bee venom varies depending on the species. The bee species mainly responsible for human envenoming are Apis mellifera melifera and Apis mellifera ligustica in Europe or Apis mellifera scutellate in Africa. Africanized bee hybrids inhabit both Americas. They are a genetic mix of the honeybee from Africa and they spread across the Americas. Data relating to toxic envenoming by species other than A. mellifera are rare [17]. Moreover, the composition of apitoxin depends on bee age, geographical localization, seasonal changes and social condition. Especially, the level of mellitin, apamin, hyaluronidase or PLA2 is very susceptible to variability. In addition, the methods collecting bee venom influence the content of volatile components. During bee venom collecting by electrical stimulation, histamine can disappear [17,18,19,20,21].

Bee venom contains several biologically active molecules such as peptides, bioactive amines and enzymes, which have an advantageous potential in healing the wounds. Bee venom contains different peptides including melittin, apamin, adolapin, secapin and its isomers (i.e., secapin-1 and -2), procamine, tertiapin and mast cell degranulating peptide (MCD-peptides) [22,23].

Peptides are the main components of bee venom. The concentration of small proteins and peptides is about 48–50% in dry venom. Among these peptides, melittin especially plays an important role in inducing reactions associated with bee stings. Melittin is a 26-amino acid peptide and constitutes the main biological active component in bee venom. Its percentage content in dry bee venom is about 50% [24]. It possesses an amphipathic property, due to the carboxyl-terminal region of the peptide, which is hydrophilic and the amino-terminal region which is hydrophobic. The amphipathic property of melittin allows it to be inserted into membranes by disrupting the phospholipid bilayers. Melittin induces membrane permeabilization and lyses of the cells. Depending on the dose, melittin induces transient or stable pores. In the case of the transient pore, the membranes are permeable for ions. In the case of the stable pore, the membranes are permeable for large molecules like glucose [25]. Melittin also induces the formation of pores responsible for its hemolytic, antimicrobial, antifungal action. At low doses, it exhibits anti-inflammatory, antibacterial, antifungal, antiviral effects and increases capillary permeability by increasing the blood circulation. However, at high doses melittin initiates local pain, itching, inflammation [26,27]. Apamin is the second very important active peptide in bee venom. It accounts for 1% of dry bee venom. It is a polypeptide which consists of 18 amino acids with two disulfide bridges. Apamin exhibits an anti-inflammatory, antinociceptive effect and increases the defense capability. It crosses the blood-brain barrier and affects the nervous system. In the vascular wall, apamin inhibits vascular smooth muscle cell proliferation and migration via the Akt and Erk signaling pathways. It inhibits PDGF-BB-induced phosphorylation of Akt and Erk1/2. PDGF, produced by activated macrophages, vascular smooth muscle cells (VSMCs) and endothelial cells or released from platelets in thrombi, plays an essential role in the abnormal proliferation and migration of VSMCs. Five different dimeric isoforms have been described including PDGF-AA, PDGF-AB, PDGF-BB, PDGF-CC and PDGF-DD. Among the isoforms of the PDGF family, the PDGF-BB-treated proliferation of VSMCs has been well demonstrated. Apamin has been found to suppress the G0/G1 cell cycle by the PDGF-BB signaling pathway and thus plays a key role in the prevention of vascular proliferation and migration constituting a promising candidate for the therapy of atherosclerosis. Apamin plays a key role in the inhibition of vascular smooth muscle cell (VSMC) proliferation and migration by the PDGF signaling pathway by preventing atherosclerosis [16,28].

Adolapin is an important component of bee venom. It is a polypeptide with 103 amino acid residues. It occurs at a concentration of 1% in dry bee venom (BV). Adolapin comprises 2–5% of the peptides in bee venom. Adolapin exhibits anti-inflammatory, antinociceptive and antipyretic effects by blocking prostaglandin synthesis and inhibiting the cyclooxygenase activity. It inhibits lipoxygenase from human platelets and may cause an analgesic effect [29,30,31]. Secapin is another polypeptide composed of 25 amino acids containing a large proportion of proline and one disulphide bridge. It is a non-toxic polypeptide which comprises only 0.5% of dry bee venom. Lee et al. showed that secapin (AcSecapin-1) from the bee venom of the Asiatic honeybee exhibits antifibrinolytic, anti-elastolytic, and antimicrobial activities [32,33]. Procamine is the next polypeptide in bee venom, which inhibits the activity of proteases like trypsin, chymotrypsin, plasmin and thrombin, thus decreasing inflammation. Tertiapin is a 21-amino acid bee venom peptide. It contains two disulphide bridges and a C-terminal residue in an amidated form. It belongs to neurotoxin peptides like apamin. Tertiapin consists of a very minor component of bee venom, comprising <0.1% of the BV dry weight. Tertiapin blocks potassium channels in the human body. It is used as a potassium channel modulator [34,35]. The mast cell degranulation peptide (MCDP) is chemically similar to apamin and comprises 22 amino acids and contains two disulfide bridges. MCDP is known as peptide 401. Its amount in bee venom is about 2–3% of dry api-toxin [27]. It exhibits immunological and pharmacological activities. It shows anti-inflammatory activity at higher concentrations, but in low concentration it plays a role as a strong mediator of mast cell degranulation and histamine release. MCDP shows an analgesic and nociceptive effect [16]. Bee venom also possesses biologically active amines like histamine, epinephrine, dopamine, norepinephrine. Bee venom contains enzymes like phospholipase A2, hyaluronidase, alfa-glucosidase, acid phosphatase, acid phosphomonoesterase, lysophospholipase. Phospholipase A2 is an enzyme hydrolyzing phospholipids and at the same time is a very strong allergen. This exhibits inflammatory effects, destroys phospholipids and disrupts the cell membrane. The concentration of phospholipase A2 in a sting is 0.23 nM. In dry venom, the percentage content is about 12–15%. Phospholipase B exhibits detoxifying activity. Hyaluronidase catalyzes the hydrolysis of hyaluronic acid by attacking the tissue hyaluronic acid polymers. Hydrolyzed hyaluronan fragments exhibit the proinflammatory, proangiogenic and immunostimulatory action. As bee venom hyaluronidase increases the capillary permeability and catalyzes the protein hydrolysis, it penetrates better into tissues [36].

Bee venom also contains dipeptidylpeptidase IV (Api m 5), Api m 6—a new bee allergen exists as four isoforms of 7190, 7400, 7598, and 7808 d, respectively (Api m 6), CUB serine protease (Api m 7), icarapin (Api m 10), major royal jelly proteins (MRJPs 8 and 9) [37,38,39,40,41].

Bee venom also contains components other than peptides including carbohydrates and free amino acids and minerals. Among carbohydrates, there are glucose and fructose in bee venom. Among amino acids, the main components are γ-aminobutyric acid and B-aminoisobutyric acid. Bee venom also contains minerals such as magnesium, calcium and phosphor. Moreover, bee venom contains volatile compounds including pheromones, which are represented by complex ethers. Isopentyl acetate and (*Z*)-11-eicosen-l-ol are pheromones that warn bees of danger and stimulate the stinging reaction [16,21,26].

## 3. Therapeutic Action of Bee Venom on Wounds

Bee venom possesses antioxidant, antimicrobial, antifungal, antiviral, anti-inflammatory and analgesic properties, so it might be a therapeutic agent in treatment of wounds. The antioxidant activity of bee venom is caused by the inhibition of the production of superoxide anion and hydrogen peroxide production in human neutrophils [16]. Moreover, bee venom was found to decrease the levels of reactive oxygen species (ROS) in animal blood and wounds, thus accelerating wound healing as reactive oxygen species lead to widespread cellular damage and to impaired neovascularization [11]. In addition to antioxidant activity, bee venom possesses an antimicrobial effect due to the content of melittin and secapin. Melittin exhibits the antibacterial effect against Gram-positive and Gram-negative bacteria, due to the formation of cell membrane channels [42]. Secapin directly binds to the bacteria cell walls, resulting in antimicrobial action [32]. As it was reported by Park and Lee, bee venom exhibits antifungal property via an apoptosis mechanism [43]. Additionally, the major compound of bee venom—melittin presents antiviral activity. The mechanism of melittin antiviral action is based upon the interferon type I stimulation and in this way, the inhibition of viral replication in the host cell. The conducted studies confirmed that melittin has antiviral activity against vesicular stomatitis virus, influenza A virus and herpes virus [44].

Anti-inflammatory and antinociceptive properties of bee venom are connected with decreased of expression of COX-2 and PLA_2_ and a decreased expression of TNF-α, IL-1, IL-6, NO, as well as intracellular calcium. Melittin, adolapin and tertiapin are responsible for the anti-inflammatory and analgesic effect of bee venom. Melittin and adolapin inhibit prostaglandin synthesis, hence bee venom inhibits cyclooxygenase and lipoxygenase activities [16]. Tertiapin exhibits anti-inflammatory activity by blocking potassium channels [45]. Moreover, MCDP possesses anti-inflammatory activity which was found in animal models. Many researchers indicate that BV injections lead to initial nociceptive action and prolonged antinociceptive effects [16].

Pharmacological activities of bee venom allow to use bee venom in wound healing. Multiple molecular mechanisms of bee venom explain the impact of bee venom on the mentioned process. Various growth factors and cytokines are involved in each phase of the wound healing process. These active biomolecules are secreted by inflammatory cells, keratinocytes and fibroblasts. The study conducted by Han et al. confirmed that bee venom had a significant wound-healing activity associated with decreasing levels of TGF-β1, fibronectin and VEGF [46]. TGF-β1, fibronectin and VEGF play a key role in angiogenesis, endothelial cell proliferation, smooth muscle cell growth and wound healing. The results of the conducted study indicated that bee venom effectively inhibits the accumulation of ECM and angiogenesis. According to the results of Han S. et al., bee venom decreased TGF-β1, fibronectin, and VEGFmRNA levels and increased the collagen type I mRNA level. Collagen type I is responsible for the tensile strength of wounds. Histological analyses indicate that collagen type I enhances the wound healing process [46]. The repair capacity of bee venom evaluated in an animal model was found to be satisfactory since the wound size was reduced.

Topical application of bee venom has an impact on reepithelization. The mentioned process involves the restoration of an intact epidermal barrier. Necessary for wound epithelialization is the keratinocytes migration. Hang et al. showed the effects of bee venom on keratinocyte migration in vitro. The distance of human epidermal keratinocytes migration after 48 h incubation was increased in a group treated with bee venom. This result indicates that bee venom stimulates the proliferation and migration of human epidermal keratinocytes. Moreover, the mentioned study indicates that bee venom decreases the expression of IL-8 and TNF-α in human epidermal keratinocytes from 24 to 74 h after BV treatment. These cytokines are secreted to suppress the proliferation of keratinocytes [47]. Toxic substances stimulate the expression of TNF-α and IL-8. These cytokines are secreted especially during the infection of wounds. This study shows that BV in a concentration below 100 µg/mL is not cytotoxic and its topical application accelerates cell regeneration and wound treatment [24]. Additionally, bee venom inhibits MMP-1 and MMP-3 production. MMP-1 is known as interstitial collagenase and supports the migration of keratinocytes and breaks down collagen type I, II, III and VI. MMP-3 known as matrix metalloproteinase-3 or stromelysin-1 is responsible for the hydrolysis of components ECM. Therefore, the inhibition of matrix metalloproteinase production prevents collagen damage [48].

## 4. Therapeutic Action of Bee Venom in Diabetic Wounds

Impaired diabetic wound healing involves pathophysiological mechanisms and constitutes a major problem in diabetic patients. Many studies indicate that bee venom can be used in diabetic patients because it significantly restores the levels of inflammatory cytokines, growth factors, and reduces the level of free radicals and accelerates wound closure [11,49,50]. Moreover, Badr et al. confirmed that bee venom therapy affects all phases of the wound healing process [11]. The impaired wound healing in diabetic patients is connected with the reduced expression of TGF-β and VEGF, the significant elevation caspase activity and the upregulated expression of ATF-3 (activating transcription factor -3) and iNOS (inducible nitric oxide synthase). Bee venom injected subcutaneously to diabetic mice for 15 days in a dose of 50 µL upregulated the expression of TGF-β and VEGF. Bee venom restores the levels of TGF-β and VEGF, which regulates different phases of wound healing. TGF-β is released by platelets in the early stage of the healing process and stimulates the chemotaxis of inflammatory and immune cells to the wounds, enhances extracellular matrix deposition and influences the granulation tissue formation. Additionally, TGF-β plays a crucial role in the remodeling process. At this stage, TGF-β supports the replacement of collagen type III with collagen type I and stimulates reepithelization. The significant restoration of the expression of VEGF influences the hemostasis, proliferation and remodeling phases. VEGF is responsible for stimulating platelet aggregation and makes the blood clot protect the environment of wounds, stimulates the proliferation of endothelial cells, activates angiogenesis and regulates the collagen fibers production and transforms collagen fibers into scar [10]. Summing up, bee venom restores the levels of TGF-β, and VEGF regulates different phases of healing. It has also been reported that bee venom accelerates the healing process in experimental animals by impairing the caspase-3, caspase-8, caspase-9 activity. The activity of caspase-3, -8, -9 was studied using a fluorometric protease assay. The conducted study indicated that the caspase cascade was decreased by therapy with bee venom. Caspase cascades play a crucial role in the induction of apoptosis and constitute an important factor in the impaired wound healing process. Due to inhibiting caspase-3, -8, -9 activity, bee venom stimulates endothelial progenitors cells in tissues. This study also revealed that bee venom treatment decreased the expression of ATF-3 and iNOS. Thus, bee venom reduces oxidative stress and decreases the level of free radicals. The increased activation of ATF-3 and iNOS leads to enhance oxidative stress and it is responsible for the prolonged wound healing process in diabetic mice. Therefore, it limits prolonged inflammation and accelerates wound healing in BV-treated diabetic mice. The regulated expression of ATF-3 and iNOS in diabetic mice by their impact on oxidative stress improves cellular differentiation and wound remodeling. Furthermore, the subcutaneous injection of bee venom restores the levels of the pro-inflammatory cytokines such as IL-1β, IL-6, TNF-α, released early during the healing process, and they play a key role as effectors for keratinocytes and fibroblasts stimulating epithelialization [11]. Further research on the influence of the subcutaneous injection of bee venom on the wound healing process in an animal model has been studied by Hozzein et al. [49]. In this study, bee venom was subcutaneously injected at the dose of 50 µL, for 15 days into the wounded area in diabetic mice. These results indicated that bee venom increased the content of collagen type I and β-defensin-2 (BD2) in diabetic wounds. Collagen is necessary for the formation of granulation tissue, thus BD2 is a very important factor to regulate the wound closure. Moreover, β-defensin-2 is a factor of inflammation, re-epithelization and angiogenesis. BD2 increases the production of proinflammatory cytokines and stimulates keratinocyte migration and proliferation. In this way, bee venom stimulates the wound healing process in diabetic wounds. Moreover, the therapy with bee venom decreased apoptotic macrophages which resulted in increasing the phagocytic index. In addition, bee venom activates angiogenesis via recovering Ang-1/Tie-2 signaling and activates the expression of Nrf2, beta-defensin-2. Tie-2 is an endothelial specific receptor tyrosine kinase which plays a key role as a regulator of wound healing. Angiopoietin-1 and angiopoietin-2 bind the Tie-2 receptor and induce angiogenesis. The connection of Ang-1 to Tie-2 in endothelial cells leads to the production of PDGF and the recruiting of smooth muscle cells to new capillaries. Impaired wound healing is caused by disrupting Ang-1/Tie-2 signaling. Additionally, the nuclear E2-related factor is an agonist ligand to Tie-2. After the subcutaneous injection of BV, the levels of Ang-1 Nrf2 were restored in wounds. Thus, Ang-1 and Nrf2 have an impact on neovascularization and accelerate the wound healing process. The molecular mechanism of bee venom action on neovascularization is connected with the expression of CD31. CD31 is a marker of neovascularization and angiogenesis in injured tissues. The expression of antigen CD31 occurs on the blood vessel endothelium. It is a protein that takes part in the wound healing process. Treatment with bee venom enhances expression of CD31 and in this way activates macrophage recruitment in bed wounds. Macrophages play a huge role in the healing process, as they take part in phagocytosis and influence the release of mediators: TGF-β, PDGF, TGF-α, FGF, Il-1, Il-6, TNF-α, which control the inflammatory process and modulate epithelialization and angiogenesis. An impaired healing process in diabetic wounds is connected with the reduction activity of enzymes such as: glutathione peroxidase (GSH-Px), manganese superoxide dismutase (MnSOD) and catalase (CAT). Many researchers indicate that oxidative stress and a large production of ROS delay the repair process. The therapy with the subcutaneous injection of bee venom protects against oxidative stress by activating antioxidant enzymes [49]. Deveci et al. indicate that oxidative stress is responsible for decreased keratinocyte proliferation and their migration and increased apoptosis [50].

Another study indicates that bee venom is a promising therapeutic agent in wound dressing. Amin and Abdel-Raheem (2014) revealed that bee venom-loaded wound dressing composed of polyvinyl alcohol (PVA), chitosan and bee venom enhances the healing of wounds in diabetic rats. In the case of wound dressing with a concentration of bee venom at 3 and 4% the researchers obtained a closure of wounds at 84 and 91%, respectively, after 14 days of treatment [12]. Moreover, the scar was clean, smooth and was not contaminated. In the described experimental study the content of hydroxyproline in wound tissues was found to be increased. The hydroxyproline is a marker of collagen content. Hydroxyproline is the end product of collagen breakdown, hence it is used in the quantity assessment of collagen in tissues. Increasing production of collagen is necessary to create an extracellular matrix in the stage of proliferation. Badr et al. also confirmed that bee venom treatment significantly increased collagen content in the extracellular matrix during the proliferative phase [11]. Amin and Abdel-Raheem (2014) [12] indicated that a bee venom-loaded dressing increases the glutathione level in wound tissues of diabetic rats. Bee venom at a concentration of 4% as a component of wound dressing showed antioxidant activity and exhibited the beneficial action in the wound repair process. Furthermore, the therapy with bee venom-loaded hydrogel may bring significant advantages in the case of skin wounds under hyperglycemia. Glutathione plays a key role as a scavenger of free radicals and protects against oxidant damage in tissues. The antioxidant role of glutathione is based on its reactivity with free organic radicals and allows for the regeneration of damaged molecules. BV, PVA and chitosan in combination possess the anti-inflammatory effect through the inhibition of the production of IL-6. Bee venom at a concentration of 4% reduced carrageenan-induced rat paw edema at similar degree as 1% diclofenac [12].

Other studies showed that bee venom at a concentration of 6% with chitosan film demonstrates a similar anti-inflammatory effect like indomethacin. BV in one topical formulation reduced PGE_2_ in the serum of rats [13]. Thus, bee venom-loaded wound dressing with PBA and chitosan accelerated wound healing. The implementation of bee venom into dressing as a therapeutic strategy demonstrates that the topical application of BV can promote cell regeneration and wound treatment. Table 1 and Table 2 summarize the effect of bee venom’s compounds on healing wounds process and the molecular mechanisms of bee venom on the skin wound healing process.

## 5. Allergic Reaction to Bee Venom

Bee venom may induce allergic reactions following the sting. Allergic reactions may occur in the skin, the respiratory track, the cardiovascular system, and the gastrointestinal system. Moreover, an anaphylactic reaction can lead to cerebral or myocardial ischemia [52,53]. Bee venom contains 12 main allergens, which are multiple protein allergens possessing an enzymatic activity and being responsible for an allergic response. Among the main allergens there are: phospholipase A2 (PLA2) called Api m1, hyaluronidase called Api m2, acid phosphatase called Api m3, melittin called Api m4, dipeptidyl-peptidase IV called Api m5, Api m6 which was w new bee venom allergen, protease called Api m7, carboxyl-esterase called Api m8, carboxyl-peptidase called Api m9, incarapin called Api m10, major royal jelly protein called Api m11 and vitellogenin called Api m12 [38,54,55,56]. Among the most important allergens mentioned are Api m1, Api m2, Api m3 and Api m4. Phospholipase A2 is the most allergenic and immunogenic protein. Hyaluronidase is known as spreading factor because it takes part in the distribution of apitoxin in the body. Acid phosphatase is capable of releasing histamine from sensitized human basophils. Mellitin is responsible for inducing minor allergic reactions [57,58]. Among people who are hypersensitive to bee venom, 97% possess specific antibodies directed against phospholipase A2, while 50% of allergic patients have sIgE for hialuronidase. In people with confirmed bee venom allergy, a specific IgE for acid phosphatase among 60% patients and 25–50% of them possess a specific IgE for melittin [54]. These allergens are responsible for allergic reactions, but bee venom also contains allergens which are involved in IgE-independent reactions, such as the bradykinin (BK) mediator, and in this way give various anaphylactic symptoms [59,60,61]. A non-immune mediator can be produced after induction by melittin. Melittin constitutes of an activator of PLA2 that can imitate BK’s effects on tracheal tone [27]. Moreover, MCD peptides can exchange disulfate between IgE on the mast cell surface and in this way MCD may inhibit the release of histamine. Due to this action, MCD can play a pivotal role as an anti-allergic agent [16]. The reaction to the bee sting may occur as a normal reaction. The normal reaction after bee sting manifests by appearing pain, redness and edema. The pain persists for 24 h. In a large local reaction of the bee sting, pain can persist for several days and the area of redness and swelling may cover a diameter greater than 10 cm. This reaction is caused by the late phase of an allergic reaction. In these cases, topical corticosteroids and H-1 and H-2 blockers are applied orally. However, in patients with allergic reactions to bee venom, there not only large local reactions but also systemic symptoms including anaphylactic shock may occur. Patients with asthma and allergic rhinitis and allergy to bee stings in the family may experience a systemic reaction [62]. Patients may suffer from symptoms of the respiratory, digestive, circulatory and nervous systems. Among allergic patients, some could occur systemic urticaria, pruritus, angioedema, vomiting or diarrhea [63,64]. In practice, the classification of the severity of the systemic reaction was developed by Ring and Messmer [65]. This classification divides the severity of the systemic reaction into four degrees. The first degree includes generalized cutaneous manifestations and angioedema. The second degree includes symptoms of the respiratory, circulatory and digestive systems. The third degree includes an anaphylactic shock and loss of consciousness. The last degree manifests itself through cardiac arrest and apnea. In anaphylaxis, the first choice of action should be administered intramuscular adrenaline. Then, the stinger should be removed. Some authors indicated that the first line of action to scrap off and take out the sting in a way that prevents the venom from entering the body. However, it is safer to administer adrenaline first [66]. In addition, short-acting β_2_ agonists are inhaled. Then, H1- and H2- blockers and corticosteroids are given [66,67,68].

Venom immunotherapy treatment (VIT) is the preventive treatment of hymenoptera venom anaphylaxis. VIT provides long-lasting immunoprotection against severe reactions to subsequent stings. Immunotherapy consists of increasing the doses of an allergen (in this case bee venom) over a period of time in order to obtain tolerance to this allergen. VIT can be conducted with different venom products (purified and non-purified, aqueous or depot). Bee venom products can be administered by the subcutaneous or sublingual routes. The effective immunotolerance of allergens results from the different mechanisms. The desensitization of mast cells and basophils, inhibition of innate lymphoid cells, the activation of regulatory T cells (which caused the increasing level of IL-10 and TGF-β), immunoglobulin cell-switch to IgG4 and IgA induced by regulatory T cells cytokines, are responsible for restoring the immunotolerance to bee venom. VIT should be performed under medical care due to the possibility occurring due to an allergic reaction [56,58,69]. A study found that in some cases, during the administration of maintenance immunotherapy, a patient had an allergic reaction. Anaphylactic shock in the 31-year-old woman in her premenstrual period appeared despite the lack of complications in the course of initiating immunotherapy and the first six maintenance doses. Anaphylactic shock was most likely influenced by the premenstrual period and withdrawal of oral contraceptives [70].

It is worth drawing attention to bee venom therapy in patients treated with angiotensin converting enzyme inhibitors and β-blockers. It was confirmed that angiotensin converting enzyme inhibitors and β-blockers used in therapy increase the risk of large allergic reaction after stinging. Therefore, before the therapy of bee venom, the problem of potential interactions between bee venom with medications taken should be considered [71].

Due to the allergic reaction to bee venom in patients, before proper bee venom therapy a basic diagnostic could be applied such as: skin test as a prick test and assays specific IgG4 levels. However, in individual situations, in case the negative results of prick test or specific IgG4 levels additional tests as component-resolved diagnosis, cell-based assays can be used. Moreover, in risk assessment systemic allergy reactions (SAR) tryptase assays should be conducted. Currently, in the diagnosis of allergy to bee venom there are conducted assays of concentration of specific IgG4 for phospholipase A2 and lymphocytes B and T [54].

## 6. Conclusions

The presented studies reveal that bee venom treatment influences all the phases of the healing process. Bee venom can be applied subcutaneously or topically in the area of wounds. Based on research conducted on animal models, the bee venom seems to be promising the potential repair agent of wounds in diabetic patients. Bee venom stimulates insulin release and lowers glycemia in the animal model. Moreover, bee venom inhibits prolonged inflammation, reduces levels of proinflammatory cytokines and reduces oxidative stress. This plays an important role in restoring delayed wound healing as it decreases caspase-3, caspase-8 and caspase-9 activity. Additionally, treatment with BV regulates the level of TGF-β, which is responsible for keratinocytes migration. This also restores the VEGF level which is a crucial factor for tissue repair.

Bee venom restores the expression of ATF-3 and iNOS, which play a crucial role in damaged tissue remodeling. The presented molecular mechanisms confirm that bee venom accelerates the wound healing process. In the light of the information presented, it will be justified to conduct research on the possibility of using a regenerative system containing a polymer with bee venom, similarly to the case of a polymer dressing with propolis [72,73]. Further studies are needed to evaluate the advantages and disadvantages of treating wounds with bee venom. Despite that the apitoxin may cause an allergic effect, therapy with bee venom brings a satisfactory effect, and therefore a venom dressing should be developed in order to minimize the systemic side effects. The therapeutic value of bee venom is huge due to antioxidant, antimicrobial, antifungal, antiviral, anti-inflammatory and analgesic properties, so it might be an attractive therapeutic agent in the treatment of wounds.

## Figures and Tables

**Table 1:** Effect of compounds of bee venom on healing wounds.

Component	Effect on Healing Wounds	References
Adolapin	Antinociceptive action; antibacterial action; anti-inflammatory action; inhibition of cyclooxygenase activity and PLA2	[46]
Apamin	Antinociceptive action; anti-inflammatory action; antibacterial action	[46]
Hyaluronidase	Selectively degrade tissue hyaluronic acid polymers; increases the capillary permeability	[51]
Melittin	Induces membrane permeabilization by reorganizing phospholipid bilayer; increases blood circulation; anti-inflammatory, antibacterial, antifungal and antiviral activity; amphipathic properties	[26,27,46]
Peptide 401 The mast cell degranulation peptide	Anti-inflammatory activity; analgesic effect; nociceptive effect	[16]
Secapin	Antibacterial properties; antifungal properties; antifibrinolytic; anti-elastolytic	[32]
Tertiapin	Anti-inflammatory activity by blocking potassium channels	[45]

**Table 2:** Molecular mechanisms of bee venom on skin wound healing process.

Phase of Healing	Molecular Mechanism of Bee Venom	References
Hemostasis	Upregulates the expression of TGF-β and VEGF in diabetic mice	[11]
Inflammation	Limits prolonged inflammation Reduces levels of proinflammatory cytokines IL-1, IL-6, TNF-α Decreases the expression of ATF-3 and iNOS	[11]
Biosynthesis of the extracellular matrix	Increases collagen formation	[11,12]
Reepithelization	Stimulates human epidermal keratinocytes proliferation and migration; Decreases level of IL-8, TNF-α	[24]
Neovascularization and angiogenesis	Decreases activity of caspase-3, -8 and -9; Upregulates the expression of TGF-β and VEGF in diabetic mice	[11]
Remodeling	Decreases levels of TGF-β1, fibronectin and VEGF; Increases collagen-I mRNA level; Decreases expression of ATF-3 and iNOS	[11,46]
